# Severity of early diagnosed organ/space surgical site infection in elective gastrointestinal and hepatopancreatobiliary surgery

**DOI:** 10.1002/ags3.12539

**Published:** 2021-12-21

**Authors:** Jun Okui, Hideaki Obara, Gaku Shimane, Yasunori Sato, Hirofumi Kawakubo, Minoru Kitago, Koji Okabayashi, Yuko Kitagawa

**Affiliations:** ^1^ Department of Surgery Keio University School of Medicine Tokyo Japan; ^2^ Department of Preventive Medicine and Public Health Keio University School of Medicine Tokyo Japan

**Keywords:** abdominal abscess, anastomotic leak, digestive system surgical procedures, pancreatic fistula, surgical wound infection

## Abstract

**Background:**

Organ/space surgical site infection (SSI) is a significant clinical problem. The postdiagnosis course of organ/space SSIs and the impact of its early diagnosis on clinical outcomes are yet to be clarified. Thus, we aimed to investigate the association between the timing of diagnosis and the clinical outcome of organ/space SSI.

**Methods:**

This retrospective, single‐center cohort study evaluated patients who underwent elective gastrointestinal or hepatopancreatobiliary surgery between 2016 and 2020. Clinical outcomes were compared between the early group (ie, SSI diagnosed until postoperative day [POD] 4) and normal‐late group (ie, SSI diagnosed after POD 5). The primary outcome was the final C‐reactive protein (CRP) level within 14 d after organ/space SSI diagnosis.

**Results:**

In total, 110 patients were evaluated. The median time of diagnosis was 7 d postoperatively (interquartile range, 5–9 d postoperatively). Compared with the normal‐late group, the early group included a higher proportion of patients with Clavien–Dindo grade ≥IIIb (8/21 vs 11/89, *P* = .01), higher final CRP value within 14 d after SSI diagnosis (mean, 4.49 mg/dL vs 2.27 mg/dL, *P* = .01), longer postoperative length of hospitalization (median, 45.0 d vs 33.0 d; *P* = .028), and worse 1‐y overall survival rate (74.8% vs 89.3%, *P* = .08).

**Conclusion:**

Early diagnosed organ/space SSI are originally severe and may therefore be detected earlier. Importantly, early diagnosed organ/space SSI is likely to be severe and refractory.

## Graphical abstract

The postdiagnosis course of organ/space SSIs and the impact of its early diagnosis on clinical outcomes are yet to be clarified. In this study the early diagnosed group had a higher final C‐reactive protein level within 14 d after diagnosis. Physicians should be aware that early diagnosed SSI cases are likely to be severe and refractory.

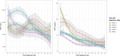

## 1 INTRODUCTION

Organ/space surgical site infections (SSIs) include various types of pivotal surgical complications, including anastomotic leakage, pancreatic fistula, biliary fistula, or intraabdominal abscess. Organ/space SSI is a significant clinical problem, as it is associated with increased mortality, prolonged hospitalization, increased medical costs,^1^ and poor oncological outcomes.^2^ Early detection and treatment are speculated to improve the outcomes of organ/space SSI, but diagnosis in the early postoperative period is challenging.^3^ A previous study reported that an anastomotic leak in colorectal surgery was diagnosed within a mean of 8.8 d postoperatively.^4^

Delayed diagnosis is mainly attributed to the fact that organ/space SSIs become apparent only after the development of clinical symptoms.^5^ Flooden et al suggested that more severe leakages cause symptoms earlier, whereas less severe leakages take longer to develop symptoms.^6^ In addition, Morks et al reported that early anastomotic leakage is associated with a higher probability of relaparotomy than late anastomotic leakage.^7^ Many predictive models using postoperative inflammatory markers have been developed for the early detection of organ/space SSIs.^8^ However, no study to date has evaluated the postdiagnosis course of organ/space SSIs that could actually be diagnosed early.

Thus, this study aimed to investigate the association between the timing of diagnosis and clinical outcome of organ/space SSI in patients who have undergone either elective gastrointestinal (GI) or hepatopancreatobiliary (HPB) surgery. In particular, we focused on daily changes in C‐reactive protein (CRP) levels after diagnosis and subsequent treatment of organ/space SSIs.

## 2 METHODS

### 2.1 Study design

This retrospective, observational, single‐center study was conducted in the Department of Surgery at Keio University Hospital, which is a tertiary care teaching hospital with approximately 1000 beds. The study protocol was approved by the Institutional Review Board of the Keio University School of Medicine (ethics approval number 20120454) and adhered to the Strengthening the Reporting of Observational Studies in Epidemiology guidelines for reporting.^9^ The need for informed consent was waived owing to the retrospective nature of the study.

### 2.2 Patient selection

The study included patients who underwent elective gastrointestinal and hepatopancreatobiliary surgery with general anesthesia between January 1, 2016, and December 31, 2020. Patients who underwent liver transplant, hernia repair, and abdominal surgery not involving the GI tract or biliary system (eg, adhesive small bowel surgery without intestinal resection, abdominal irrigation for generalized peritonitis, and laparotomy hemostasis) were excluded. Liver transplant was excluded due to the heterogeneous nature of the surgery. In addition, we excluded patients in whom only ileostomy or colostomy was performed because these surgeries have no risk of organ/space SSI. Finally, we also excluded emergency cases because they have a significantly different mechanism of postoperative intraabdominal infection.

Surgical procedures were classified according to the Japan Nosocomial Infections Surveillance (JANIS)^10^ surgical classification criteria (Table S1). For patients who underwent simultaneous multiorgan surgeries, only the primary procedure was recorded as the surgical procedure.

### 2.3 Definition of organ/space SSI

Anastomotic leakage, pancreatic fistula, biliary fistula, and intraabdominal abscess diagnosed until postoperative day (POD) 30 were considered as organ/space SSIs. Organ/space SSI was diagnosed by fellow and attending surgeons using the Centers for Disease Control and Prevention (CDC) National Healthcare Safety Network (formerly the National Nosocomial Infection Surveillance System) definitions^11^ (Table S2). The day when the CDC criteria were met for the first time was designated as the SSI diagnosis date. At Keio University Hospital, all patients with organ/space SSI patients are provided therapeutic intervention of at least antibiotic administration on the day of diagnosis. Therefore, in this study we considered the date of diagnosis as the start date of organ/space SSI treatment.

Pancreatic fistula was evaluated using the CDC definitions for all types of resection organs, instead of the more commonly used International Study Group for Pancreatic Surgery (ISGPS) criteria revised in 2016.^12^ This is because our SSI surveillance is based on the national survey. Complications were graded using the Clavien–Dindo classification,^13^ and we included only Clavien–Dindo grade ≥II organ/space SSI in the analysis because grade I does not require clinical intervention. Supplementally, grade IIIb requires intervention under general anesthesia, grade IV requires intensive care unit management with life‐threatening complications, and grade V indicates the death of a patient.^13^ In this study, Clavien–Dindo grade V was defined as death caused by organ/space SSI during the same hospitalization stay. If patients were simultaneously diagnosed with multiple types of organ/space SSIs, the only primary complication was determined as the outcome. In addition, for patients who underwent multiple surgeries during the same hospitalization and developed organ/space SSI, only the surgery that directly caused SSI was counted. Given that the presence or absence of complications is recorded on the surveillance sheet at the time of hospital discharge, patients who developed organ/space SSI after discharge could not be included.

### 2.4 Data collection

Patient data from the electronic medical records were reviewed by author J.O. Clinicodemographic data (ie, age, sex, American Society of Anesthesiologists physical status, body mass index, diabetes mellitus, albumin level, and smoking history), intraoperative data (ie, surgical procedures and estimated blood loss), and postoperative data (ie, length of hospitalization and 1‐y survival) were collected. Postoperative survival information was collected on August 31, 2021.

### 2.5 Primary outcome

Laboratory findings within 14 d after organ/space SSI diagnosis were collected. In particular, we focused on postdiagnosis inflammatory changes (white cell count [WCC] and CRP levels), and thus, we did not collect laboratory data based on the operative day. The final CRP value within 14 d after diagnosis was set as the primary outcome for the following reasons: Nakamura et al investigated the 14‐day changes in CRP among patients with persistent inflammatory, immunosuppressed, catabolic syndrome (PIICS)^14^ and established that a CRP level of 3.0 mg/dL on day 14 was the optimal cutoff for PIICS diagnosis. Inspired by this research, we investigated the changes in CRP levels over 14 d. Further, since we considered the inflammatory response in the early postdiagnosis period to be highly variable, we focused on the values on day 14 to investigate the final outcome of treatment.

### 2.6 Statistical analysis

Univariate analysis was performed to examine the background differences between organ/space SSIs diagnosed until POD 4 (early diagnosis group) and after POD 5 (normal‐late diagnosis group). There were several reasons for adopting this division. First, in our previous study the median diagnosis date of organ/space SSI was POD 6 (interquartile range, 4–9 d).^8^ Second, postoperative laboratory tests are likely to be performed on odd days in our hospital. Considering the division by odd‐numbered days, we thought it was appropriate to set POD 1–4 as the cutoff to define the early diagnosis group.

Continuous variables were presented as the means and standard deviations (SDs) and analyzed using a *t*‐test. Meanwhile, categorical variables were presented as frequencies and proportions and analyzed using Fisher's exact test. Length of hospitalization and duration of operation were expressed as median and interquartile range and was analyzed using the Mann–Whitney *U* test. As a supplement, the date of diagnosis by type of organ/space SSI, the date of diagnosis by type of surgical procedure, and final laboratory test date by the date of diagnosis were expressed as median and interquartile range and were analyzed using the Kruskal–Wallis test. Further, organ/space SSI diagnosis date was divided into 2 d and plotted the transition of WCC and CRP for 14 d after the start of organ/space SSI treatment. The reason for dividing by 2 d was that postoperative laboratory tests are likely to be performed on odd days in our hospital. In addition, a local regression line generated using the locally estimated scatterplot smoothing method with 95% confidence interval (CI) was added.^15^

The 1‐y overall survival rate was determined by generating Kaplan–Meier survival curves and then comparing these between the two groups using the log‐rank test and the Cox proportional hazard model. The hazard ratio (HR) with a 95% CI was estimated. All statistical analyses were performed using R statistical software v. 4.0.3 (R Foundation for Statistical Computing, Vienna, Austria). All *P*‐values were two‐tailed, and statistical significance was set at *P* < .05.

## 3 RESULTS

**FIGURE 1:**
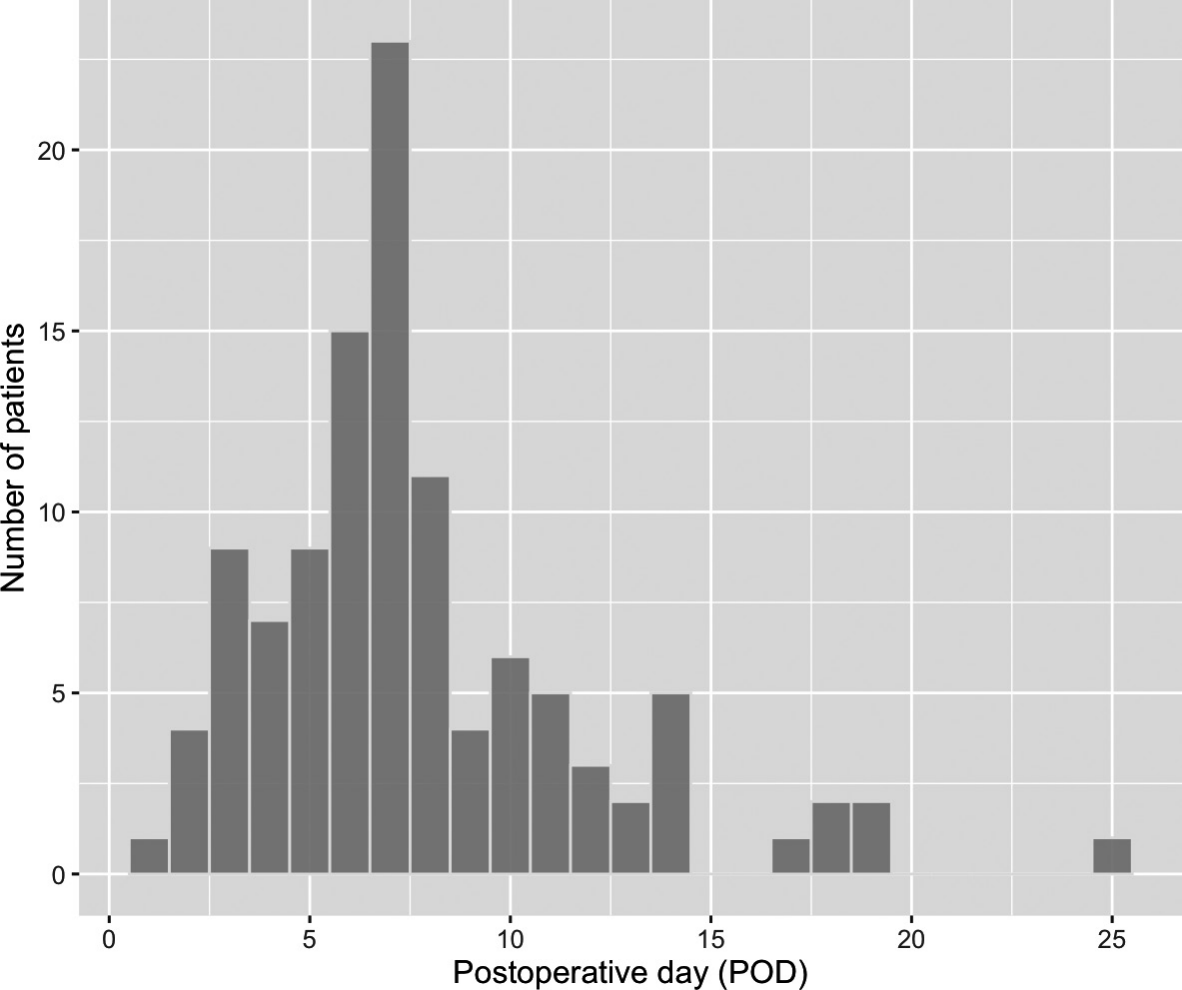
Day of organ/space SSI diagnosis. SSI, surgical site infection

Among the 3310 patients who underwent elective gastrointestinal and pancreatobiliary surgery, 110 patients (3.3%) developed organ/space SSI (anastomotic leak, n = 38; pancreatic fistula, n = 35; biliary fistula, n = 13; and intraabdominal abscess, n = 24) (Table 1 and S3). In total, 14.2%, 6.5%, and 4.8% developed organ/space SSI after pancreaticoduodenectomy, esophageal surgery, and rectal surgery, respectively (Table 1). The median time between surgery and the diagnosis of organ/space SSI was 7 d (interquartile range, 5–9 d) (Figure 1). The median date of diagnosis by type of organ/space SSI was POD 8.5 for intraabdominal abscess, POD 7 for biliary fistula, POD 6 for anastomotic leakage, and POD 7 for pancreatic fistula (Figure S1). Intraabdominal abscesses tended to be diagnosed late. The median date of diagnosis by type of surgical procedure is shown in Figure S2 and there were no significant differences among procedure types (*P* = .83).

**TABLE 1:** Organ/space SSI cases stratified by the surgical procedures

Surgical procedures	Keio University Hospital in 2016–2020 (Elective surgery)			JANIS in 2020 (Elective & emergency surgery)*		
	n	Organ/space SSI	Incidence	n	Organ/space SSI	Incidence
ESOP	294	19	6.5%	1563	111	7.1%
GAST‐D	319	10	3.1%	7370	254	3.4%
GAST‐T	99	1	1.0%	3483	239	6.9%
GAST‐O	194	2	1.0%	4932	171	3.5%
SB	226	1	0.4%	7171	220	3.1%
COLO	629	10	1.6%	46 193	1150	2.5%
APPY	35	0	0.0%	13 411	186	1.4%
REC	231	11	4.8%	15 987	883	5.5%
BILI‐L	303	14	4.6%	5067	187	3.7%
BILI‐PD	190	27	14.2%	3167	562	17.7%
BILI‐O	310	13	4.2%	3419	376	11.0%
CHOL	463	2	0.4%	27 340	189	0.7%
SPLE	17	0	0.0%	301	9	3.0%
Total	3310	110	3.3%	68 692	2,392	3.5%

Note:

Data are reported as number of patients (% are calculated on the total patients in the line).

We processed Japan nosocomial infections surveillance (JANIS) data in 2020 (available at: https://janis.mhlw.go.jp/english/index.asp. Accessed August 31, 2021).

JANIS is the national survey in Japan, in which 2418 hospitals participated. Surgical procedures are classified according to JANIS surgical classification criteria (See Table S1).

Abbreviation: SSI, surgical site infection.

* JANIS does not separate elective and emergency cases for organ/space SSI. We compared the incidence of organ/space SSI including both types of surgery in Table S4.

In total, 21 patients (19.1%) and 89 patients (80.9%) were categorized into the early and normal‐late diagnosis groups, respectively. The results of univariate analysis are shown in Tables 2 and 3. Preoperative and intraoperative variables did not differ significantly between groups, including surgical procedures and duration of operation. Notably, compared with the normal‐late diagnosis group, the early diagnosis group showed a significantly longer median postoperative length of hospitalization (45.0 d vs 33.0 d, respectively; *P* = .028) and had a higher proportion of patients with Clavien–Dindo grade ≥IIIb complications (8/21 vs 11/89, respectively; *P* = .01), and had a higher proportion of patients with relaparotomy (5/21 vs 6/89, *P* = .034).

**TABLE 2:** Preoperative and intraoperative patient characteristics

n	Organ/space SSI diagnosis date		*P*‐value
	POD 1–4 (early diagnosis group)	POD 5– (normal‐late diagnosis group)	
	21	89	
Preoperative information			
Sex = Male	17 (81.0%)	55 (61.8%)	.128
Age	68.2 (9.6)	67.3 (12.3)	.75
Diabetes mellitus	3 (14.3%)	22 (24.7%)	.394
Albumin (g/dL)	3.71 (0.48)	3.90 (0.53)	.155
BMI	22.7 (2.7)	22.4 (3.7)	.79
Past or present smoking habit	13 (61.9%)	40 (44.9%)	.225
ASA‐PS			.425
1	4 (19.0%)	30 (33.7%)	
2	15 (71.4%)	52 (58.4%)	
3	2 (9.5%)	7 (7.9%)	
Intraoperative information			
Surgical procedure (%)			.418
ESOP	4 (19.0%)	15 (16.9%)	
GAST‐D	2 (9.5%)	8 (9.0%)	
GAST‐T	0 (0.0%)	1 (1.1%)	
GAST‐O	1 (4.8%)	1 (1.1%)	
SB	1 (4.8%)	0 (0.0%)	
COLO	0 (0.0%)	10 (11.2%)	
REC	2 (9.5%)	9 (10.1%)	
BILI‐L	3 (14.3%)	11 (12.4%)	
BILI‐PD	7 (33.3%)	20 (22.5%)	
BILI‐O	1 (4.8%)	12 (13.5%)	
CHOL	0 (0.0%)	2 (2.2%)	
Laparotomy	13 (61.9%)	48 (53.9%)	.627
Estimated blood loss (mL)	33.1 (18.8)	30.1 (20.5)	.54
Duration of operation (min) (median [IQR])	425 [270‐535]	405 [297‐512]	.924

Note:

Continuous values represent mean (SD). Categorical values represent n (%).

Continuous variables were analyzed by Welch's *t*‐test, and categorical variables were analyzed by Fisher's exact test. Duration of operation was expressed in median and interquartile range, and was analyzed by Mann–Whitney *U* test.

Surgical procedures are classified according to Japan nosocomial infections surveillance (JANIS) surgical classification criteria (See Table S1).

Abbreviations: ASA‐PS, American Society of Anesthesiologists physical status; BMI, body mass index; POD, postoperative day; SSI, surgical site infection.

**TABLE 3:** Postoperative outcomes

n	Organ/space SSI diagnosis date		*P*‐value
	POD 1–4 (early diagnosis group)	POD 5– (normal‐late diagnosis group)	
	21	89	
Postoperative length of stay (day) (median [IQR])	45.0 [34.0, 73.0]	33.0 [24.0, 52.0]	.028
Postdiagnosis length of stay (day) (median [IQR])	44.0 [30.0, 69.0]	25.0 [14.0, 42.0]	.002
Type of organ/space SSI			.17
Anastomotic leakage	8 (38.1%)	30 (33.7%)	
Pancreatic fistula	8 (38.1%)	27 (30.3%)	
Biliary fistula	4 (19.0%)	9 (10.1%)	
Intraabdominal abscess	1 (4.8%)	23 (25.8%)	
Clavien–Dindo Grade			.006
II	0 (0.0%)	19 (21.3%)	
IIIa	13 (61.9%)	59 (66.3%)	
IIIb	1 (4.8%)	2 (2.2%)	
IVa	2 (9.5%)	1 (1.1%)	
IVb	2 (9.5%)	5 (5.6%)	
V	3 (14.3%)	3 (3.4%)	
Clavien–Dindo Grade IIIb or above	8 (38.1%)	11 (12.4%)	.01
Relaparotomy	5 (23.8%)	6 (6.7%)	.034
Final laboratory test date within 14 d after starting treatment (day)	13.3 (1.6)	12.7 (1.7)	.124
Final WCC (/μL)	7819 (3483)	6909 (2683)	.191
Final CRP (mg/dL)	4.49 (4.62)	2.22 (2.80)	.004

Note:

Continuous values represent mean (SD). Categorical values represent n (%).

Continuous variables were analyzed by Welch's *t*‐test, and categorical variables were analyzed by Fisher's exact test. Length of hospitalization was expressed in median and interquartile range, and was analyzed by Mann–Whitney *U* test.

In this study, Clavien–Dindo Grade V was defined as death caused by organ/space SSI during the same hospitalization stay.

Surgical procedures are classified according to Japan nosocomial infections surveillance (JANIS) surgical classification criteria (See Table S1).

Abbreviations: CRP, C‐reactive protein; POD, postoperative day; SSI, surgical site infection; WCC, white cell count.

**FIGURE 2:**
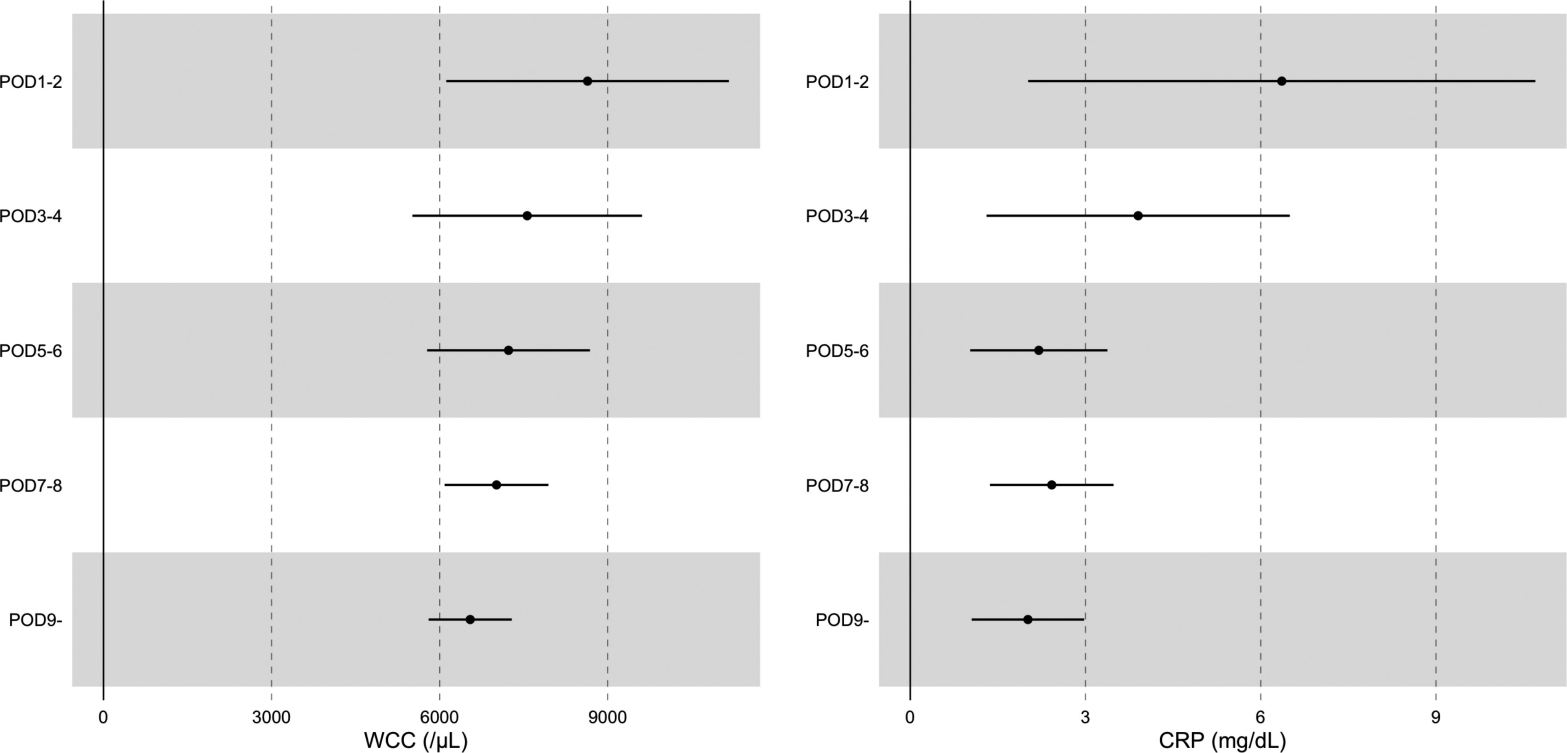
Average value of the final WCC and CRP within 14 d after organ/space SSI diagnosis classified by diagnosis date. The period represents the mean value, and the error bar represents the 95% confidence interval. CRP, C‐reactive protein; POD, postoperative day; SSI, surgical site infection; WCC, white cell count

**FIGURE 3:**
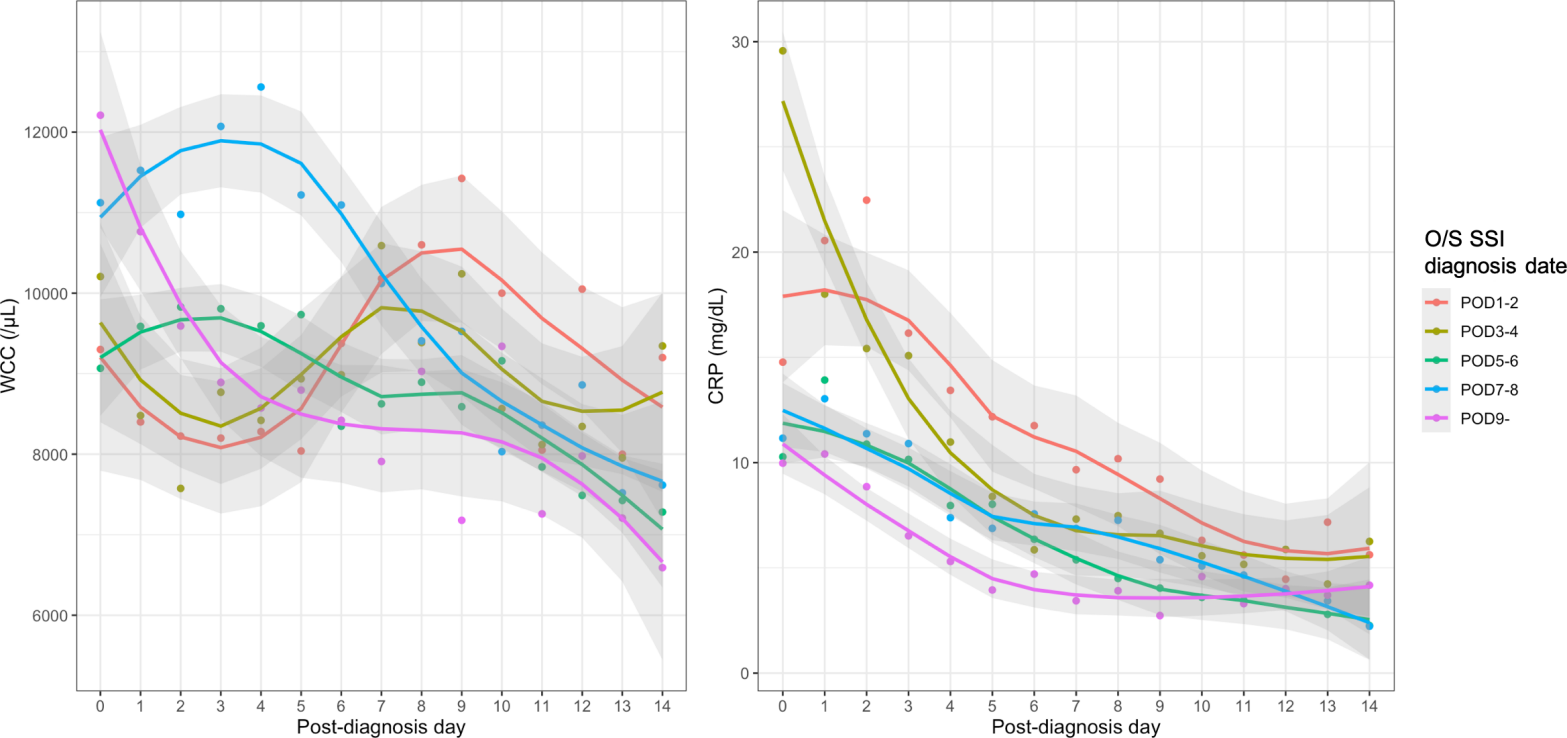
Trends in the average values of WCC and CRP within 14 d after organ/space SSI diagnosis classified by diagnosis date. CRP, C‐reactive protein; POD, postoperative day; O/S SSI, organ/space surgical site infection; WCC, white cell count

Postdiagnosis changes in CRP and WCC were followed for 14 d. As shown in Figure S3, the median final laboratory test date was above day 13 in all five groups (POD 1–2, POD 3–4, POD 5–6, POD 7–8, and POD9–). The mean final CRP and WCC values within 14 d tended to increase with an earlier diagnosis date (Figure 2). The final CRP value was significantly higher in the early diagnosis group than in the normal‐late group (mean, 4.49 mg/dL vs 2.27 mg/dL; *P* = .01, Table 3). The average CRP and WCC values tended to be higher 14 d after diagnosis in the early diagnosis group (Figure 3).

**FIGURE 4:**
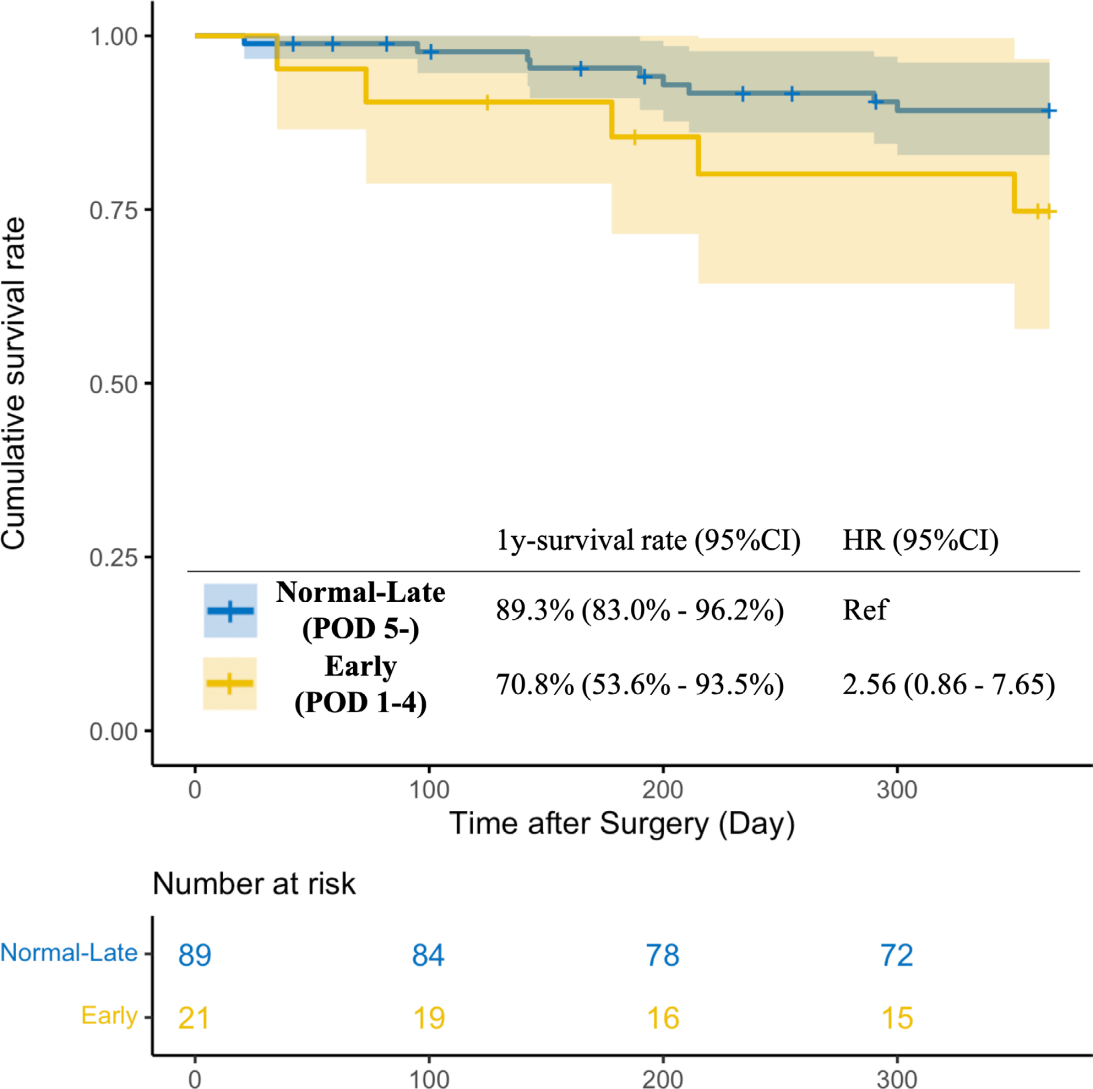
Kaplan–Meier curve for 1‐y overall survival by timing of organ/space SSI diagnosis. There are 14 events in total (five in the early diagnosis group and nine in the normal‐late diagnosis group). The hazard ratio (HR) was estimated using the Cox proportional hazard model. CI, confidence interval; POD, postoperative day; Ref, Reference; SSI, surgical site infection

The 1‐y Kaplan–Meier survival curve is shown in Figure 4. Overall, 14 events occurred in our cohort, five events in the early group, and nine events in the normal‐late group. The cause of death is shown in Table S5. In both groups, deaths caused by organ/space SSI account for the highest proportion. The overall survival rate was 86.5% (95% CI: 80.1%–93.3%) in the total population, 74.8% (95% CI: 57.8%–96.7%) in the early diagnosis group, and 89.3% (95% CI: 82.9%–96.2%) in the normal‐late diagnosis group, with no significant between‐group differences (*P* = .08). The HR for survival of the early group compared with the normal‐late group was 2.56 (95% CI: 0.86–7.65).

## 4 DISCUSSION

The postdiagnosis course of organ/space SSIs and the impact of its early diagnosis on clinical outcomes are yet to be clarified. In this study, the median time between surgery and diagnosis was 7 d (interquartile range, 5–9 d). Compared with the normal‐late diagnosis group, the early diagnosis group had a longer median postoperative length of hospitalization, a higher proportion of patients with Clavien–Dindo grade ≥IIIb complications, higher final CRP value within 14 d after organ/space SSI diagnosis, and worse overall survival rate. These findings suggest that early diagnosed organ/space SSI cases were originally severe and may, therefore, be detected earlier.

The most important clinical implication of this study is that physicians should be aware that early diagnosed organ/space SSI is likely to be severe and refractory. The early diagnosed group had a worse overall survival rate than did the normal‐late group (74.8% vs 89.3%, *P* = .08). Given that these patients showed prominent clinical manifestations from the early postoperative period, organ/space SSI may have been detected early. Notably, considering the expanded use of computed tomography imaging owing to the universal health insurance in Japan, there may have been little delays in the diagnosis. The rigorous assessment of the patients diagnosed in the early postoperative period that they truly have severe disease. These patients should be given antibiotics and undergo swift drainage, regardless of the underlying mechanism. Further, physicians should be cautious against early discharge.

Another clinical implication of this study is that the normal or late‐diagnosed organ/space SSI cases are less likely to become severe. The final CRP and WCC values within 14 d postoperatively tended to be lower in those diagnosed later. Our findings suggest that cases diagnosed beyond POD 4 under strict surveillance may not be severe cases. Patients who are diagnosed beyond POD 4 under intensive surveillance may have good outcomes. Several previous studies on colorectal surgery have focused on the timing of diagnosis. Tan et al reported the characteristics of late anastomotic leakage (LAL) diagnosed after POD 30, which included younger patients than the early anastomotic leakage (EAL) group.^16^ Flooden et al suggested that early anastomotic leakage and LAL are different entities because of their different anatomical localization of anastomotic leakage points.^6^ A previous study also reported that EAL patients are more likely to undergo relaparotomy than LAL patients,^7^ and consistent findings were obtained in this study. These findings suggest that organ/space SSI diagnosed in the early stage is severe and refractory. However, these studies were limited to colorectal surgery, and no studies that included all GI and HPB surgeries were conducted.

Many previous studies have reported that postoperative CRP elevation can predict SSI after GI surgery.^17^, ^18^, ^19^ However, although there have been reports that changes in the CRP level can begin as early as the surgery date,^20^, ^21^ no study has evaluated the trends in CRP levels by the date of organ/space SSI diagnosis. Previous research suggests a CRP level of 3.0 mg/dL on day 14 is the optimal cutoff for PIICS diagnosis, as evaluated by 14‐d changes in CRP among patients with PIICS.^14^ Similarly, we investigated the changes in CRP levels over 14 d. The results showed higher CRP values prolonged until 14 d in the early diagnosis group than in the normal‐late group (mean 4.49 mg/dL vs 2.27 mg/dL; *P* = .01). Collectively, these findings support that prolonged CRP elevation is an important indicator of severity, and thus, it is reasonable to evaluate CRP levels. Additionally, we also investigated the trends in WCC, and the early diagnosed group showed a bimodal curve. The delayed second peak occurred around day 8 after diagnosis, implying severe SSI in the early diagnosis group.

Unlike previous studies that used a combination of primary outcomes, such as in‐hospital mortality, cardiovascular complications, incisional SSI, and postoperative remote infections (eg, pneumonia, postoperative urinary tract infections),^22^, ^23^, ^24^, ^25^ our study focused on organ/space SSI, which is another strength of the present study. When a composite outcome is adopted, it is sometimes difficult to make a clinical interpretation because of the diverse treatment. There are also several options for the management of organ/space SSI; these include antibiotics, bowel rest, percutaneous drainage, and relaparotomy. Further, we focused on organ/space SSIs, due to their severity. Finally, our study has the advantage of accurate observation of organ/space SSI patients owing to its single‐center setting.

However, this study also had some limitations. First, we could not investigate whether an earlier diagnosis or intervention resulted in better clinical outcomes. We could only prove the severity of early diagnosed organ/space SSI. Further investigations on the relationship between the timing of diagnosis and outcome are warranted. Prospective studies monitoring the severity of complications and including a control group with delayed treatment are needed; however, such a design is unethical. It would be more appropriate to adjust for potential confounders, such as surgical procedures, Clavien–Dindo grade, and demographic data. However, organ/space SSI is a rare complication, and thus, this study did not have sufficient power for multivariate analysis.

Second, as described above, this study investigated overall organ/space SSI regardless of the surgical organs; as such, we could not perform a more detailed subgroup analysis according to the type of surgical procedure. For example, the frequency of organ/space SSI varied greatly (14.2% after pancreaticoduodenectomy vs 0.4% after cholecystectomy). The invasiveness of these surgeries varies greatly. Further, because organ/space SSI occurred in only 3.3% of our dataset, the sample size was inadequate to investigate the relationship between the timing of diagnosis and clinical outcomes classified by surgical organs.

Third, given the single‐center study setting, we could not externally validate our findings. Treatments for organ/space SSIs differ among facilities, and it directly affects the postdiagnosis inflammatory response. However, it is difficult to collect accurate diagnosis data, including timing, from multiple facilities. Fourth, the diagnosis date may vary depending on the organ/space SSI criteria. For example, pancreatic fistula is commonly evaluated clinically using the ISGPS criteria.^12^ However, because our SSI surveillance is based on JANIS, a national survey involving 2418 hospitals, we decided to use the CDC definitions for all types of resection organs.^11^ The incidence of organ/space SSI in this study was comparable to that of the national survey in 2020. Unfortunately, JANIS does not separate elective and emergency cases for organ/space SSI; therefore, we compared the incidence of organ/space SSI without excluding emergency cases (Table S4). However, in our previous study based on the same SSI criteria, the median time between surgery and the diagnosis of organ/space SSI was 6 d (interquartile range, 4–9 d),^8^ which is not highly different from the 7 d (interquartile range, 5–9 d) in this study. These results show that there is no significant difference between our hospital and other facilities in Japan.

## 5 CONCLUSION

Early diagnosed organ/space SSI cases are originally severe and thus may be detected earlier. Physicians should be aware that early diagnosed organ/space SSI is likely to be severe and refractory. For these patients, antibiotics should be selected empirically, drainage should be performed quickly, and clinicians should be cautious about early discharge.

## DISCLOSURE

Funding: This research did not receive any specific grant from funding agencies in the public, commercial, or not‐for‐profit sectors.

Conflict of interest: The authors have no conflicts of interests to declare regarding this study.

The study protocol was approved by the Institutional Review Board of the Keio University School of Medicine (ethics approval number 20120454). The need for informed consent was waived owing to the retrospective nature of the study.

Author contributions: Okui, Obara, Shimane, Sato, Kawakubo, Kitago, Okabayashi, and Kitagawa contributed to the study conception and design. Okui and Shimane collected the data. Okui and Sato analyzed and interpreted the data. Okui, Obara, Shimane, and Sato drafted the article. Kawakubo, Kitago, Okabayashi, and Kitagawa contributed to the critical revision of the article. All authors have read and approved the final article.

Disclosures outside the scope of this work: Author H.O. received lecture fees from Otsuka Pharmaceutical Factory Inc. Author H.O. was supported by grants from Medtronic Japan Co., Ltd., Japan Blood Products Organization, TAIHO PHARMACEUTICAL CO., LTD, W. L. Gore & Associates, Co., LTD., DAIICHI SANKYO COMPANY, LIMITED, Mitsubishi Tanabe Pharma Corporation, TEIJIN PHARMA LIMITED., KAKEN PHARMACEUTICAL CO., LTD., NOVARTIS PHARMA Co., Ltd., Medtronic Japan Co., Ltd., Japan Blood Products Organization, JMS Co., Ltd., TAIHO PHARMACEUTICAL CO., LTD, MSD KK, KAKEN PHARMACEUTICAL CO., LTD., Mitsubishi Tanabe Pharma Corporation, W. L. Gore & Associates, Co., LTD., NIHON PHARMACEUTICAL CO., LTD., Japan Lifeline Co., Ltd, Medtronic Japan Co., Ltd. JMS Co., Ltd., Japan Blood Products Organization, TAIHO PHARMACEUTICAL CO., LTD. Author Y.K. received lecture fees from CHUGAI PHARMACEUTICAL CO., LTD., TAIHO PHARMACEUTICAL CO., LTD, ASAHI KASEI PHARMA CORPORATION, Otsuka Pharmaceutical Factory Inc, ONO PHARMACEUTICAL CO., LTD., SHIONOGI & CO., LTD., Nippon Covidien Inc, AstraZeneca KK, Ethicon Inc, Bristol‐Myers Squibb KK., and Olympus Corporation. Author Y.K. was supported by grants from CHUGAI PHARMACEUTICAL CO., LTD., TAIHO PHARMACEUTICAL CO., LTD, Yakult Honsha Co. Ltd., ASAHI KASEI PHARMA CORPORATION., Ltd., Otsuka Pharmaceutical Co., Ltd., ONO PHARMACEUTICAL CO., LTD., TSUMURA & CO., KAKEN PHARMACEUTICAL CO. LTD., DAINIPPON SUMITOMO PHARMA Co., Ltd., EA Pharma Co., Ltd., Eisai Co., Ltd., Otsuka Pharmaceutical Factory Inc, MEDICON INC., Kyouwa Hakkou Kirin Co., Ltd., Takeda Pharmaceutical Co., Ltd., Toyama Chemical Co., Ltd., Astellas Pharma Inc, TEIJIN PHARMA LIMITED., NIHON PHARMACEUTICAL CO., LTD., and Nippon Covidien Inc Author Y.K. held an endowed chair provided by CHUGAI PHARMACEUTICAL CO., LTD. and TAIHO PHARMACEUTICAL CO., LTD, outside the submitted work. Author Y.K. received other compensation from ONO PHARMACEUTICAL CO., LTD., and Bristol‐Myers Squibb KK.

## Supporting information

Figure S1

Figure S2

Figure S3

Table S1

Table S2

Table S3

Table S4

Table S5
