# Supplementary figures and images for: Severity of early diagnosed organ/space surgical site infection in elective gastrointestinal and hepatopancreatobiliary surgery
Source: Ann Gastroenterol Surg. 2021 Dec 21;6(3):445–53. doi: 10.1002/ags3.12539 (PMC9130879; doi:10.1002/ags3.12539)

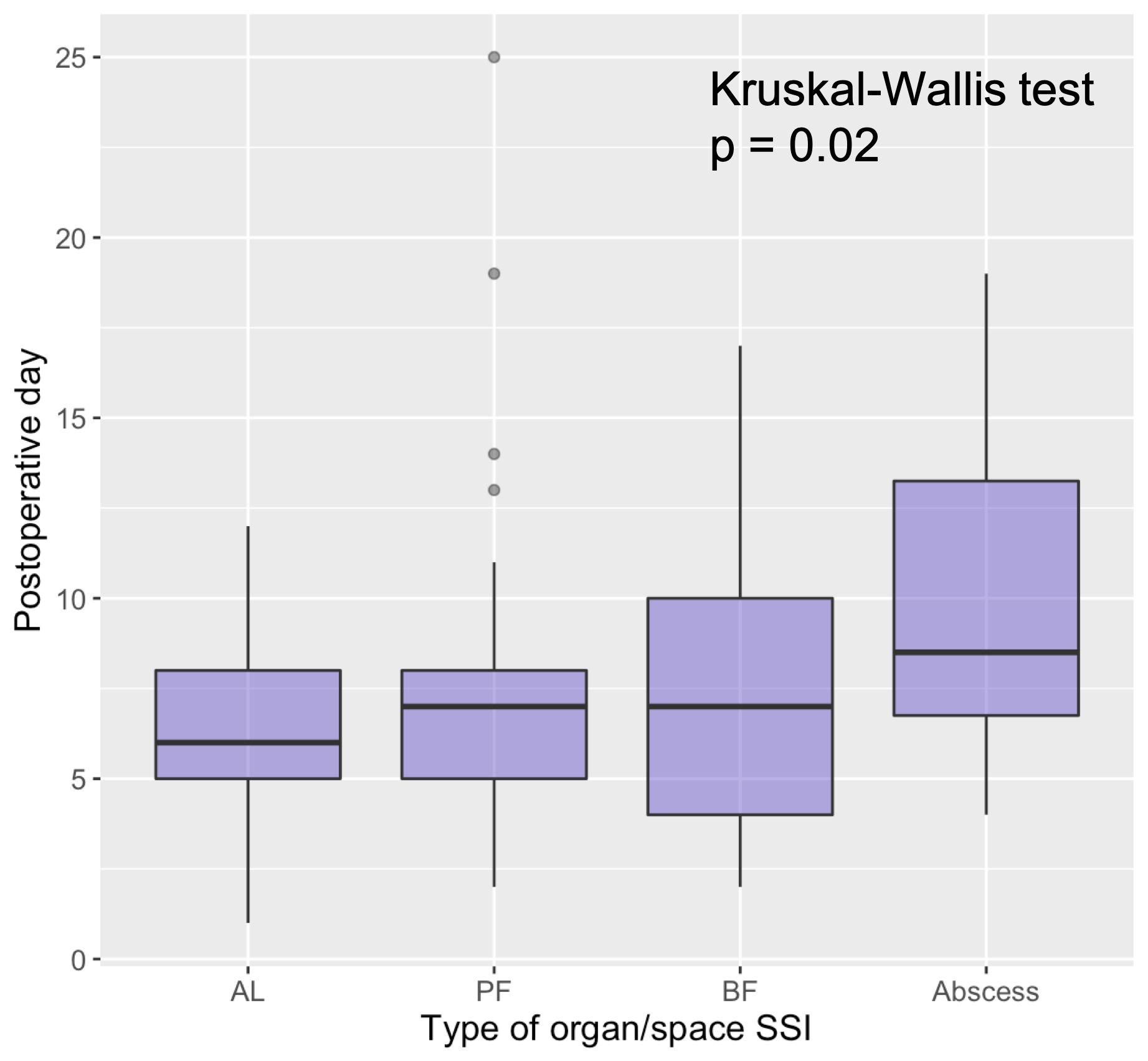

Supplement: Supplementary file 1 — Figure S1 [file AGS3-6-445-s003.tiff]

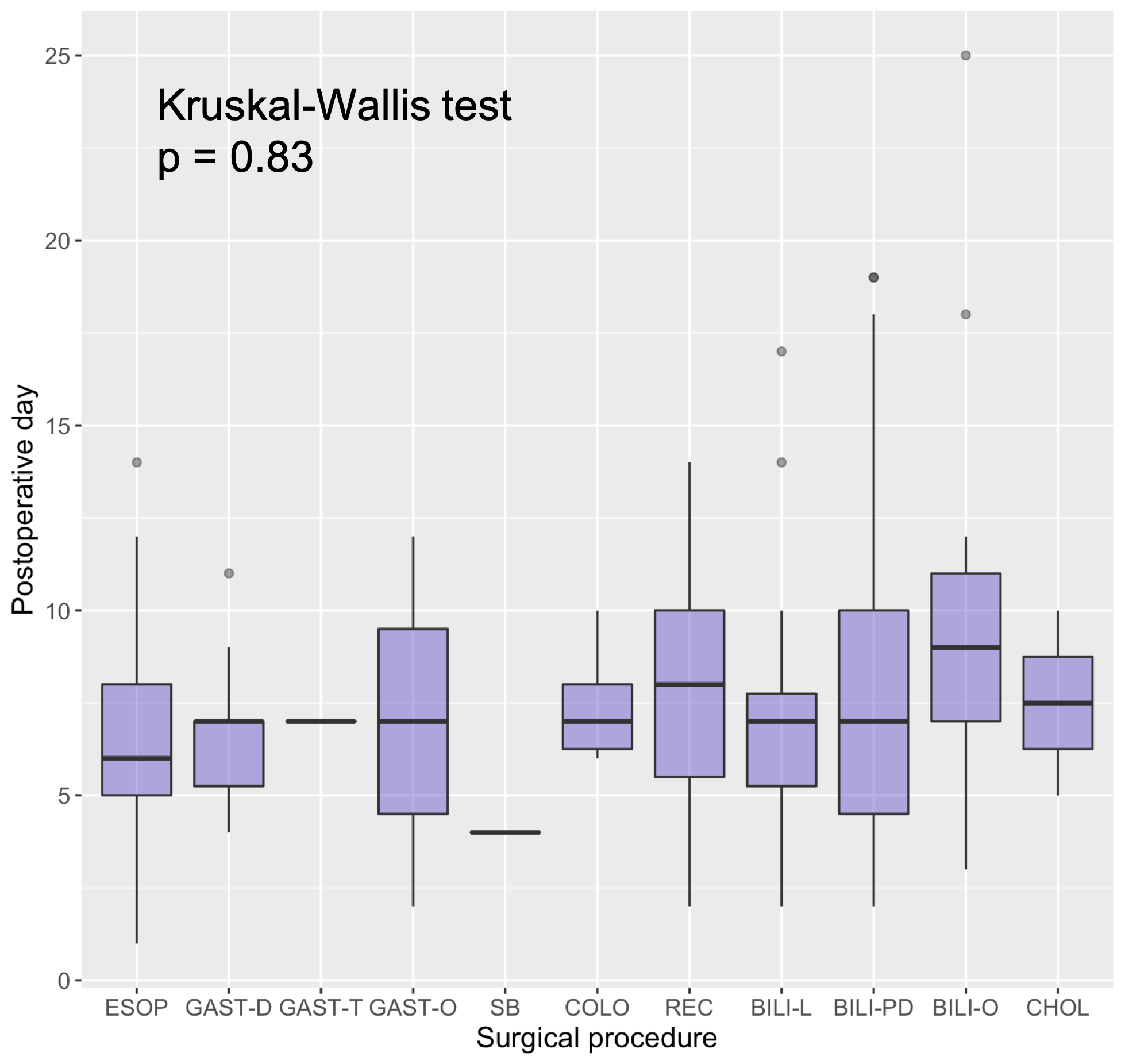

Supplement: Supplementary file 2 — Figure S2 [file AGS3-6-445-s005.tiff]

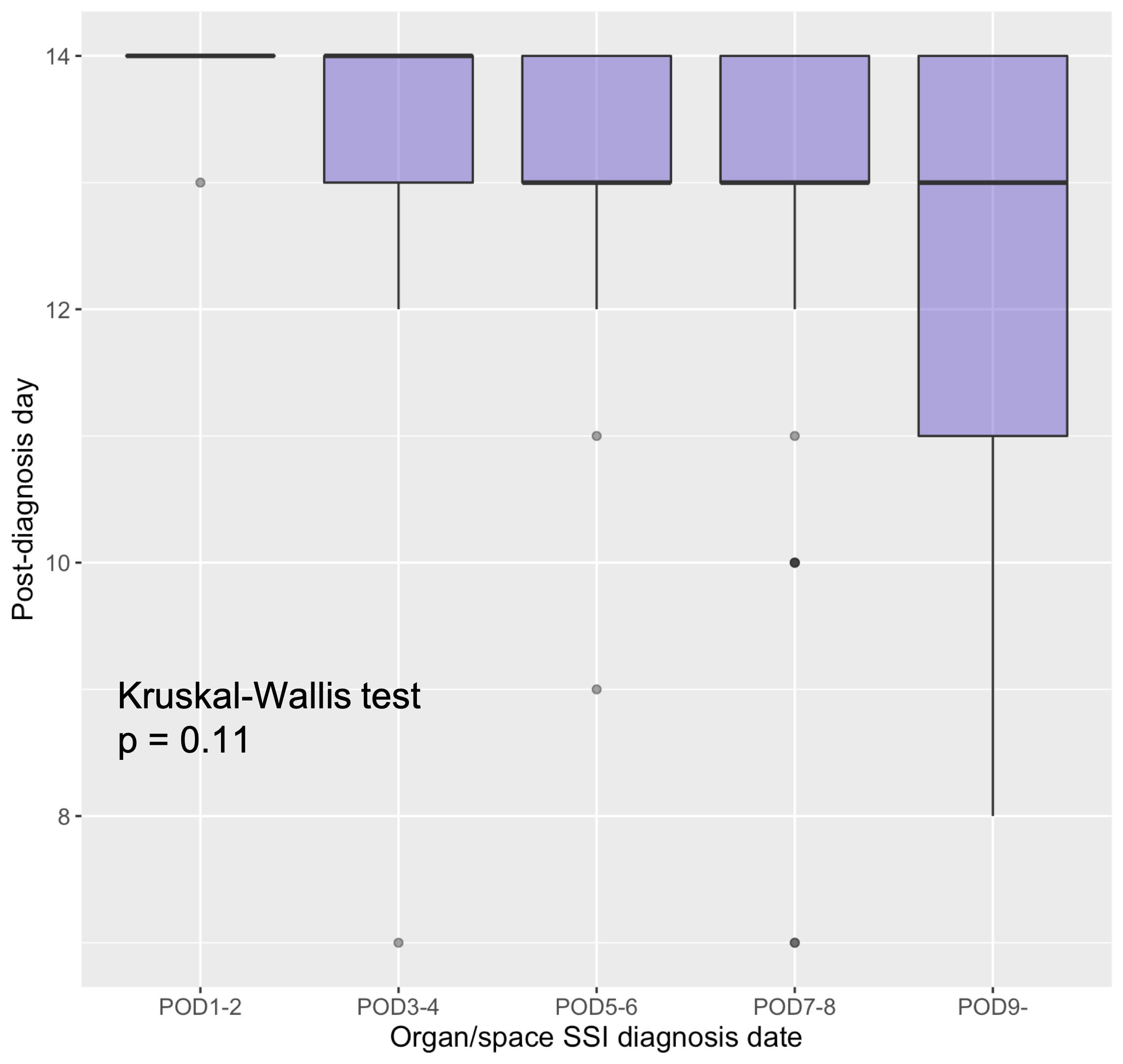

Supplement: Supplementary file 3 — Figure S3 [file AGS3-6-445-s004.tiff]
