# Supplementary material for: Severity of early diagnosed organ/space surgical site infection in elective gastrointestinal and hepatopancreatobiliary surgery
Source: Ann Gastroenterol Surg. 2021 Dec 21;6(3):445–53. doi: 10.1002/ags3.12539 (PMC9130879; doi:10.1002/ags3.12539)
Supplement: Supplementary file 4 — Table S1 [file AGS3-6-445-s007.docx]

| Supplemental Table 1. Japan nosocomial infections surveillance (JANIS) surgical classification criteria | |
| --- | --- |
| Code | Surgical procedure |
| APPY | Appendix surgery (Excluding those performed in association with other surgical procedures) |
| BILI-L | Hepatectomy without biliary reconstruction |
| BILI-PD | Pancreaticoduodenectomy |
| BILI-O | Hepatopancreatobiliary surgery (excluding hepatectomy without biliary reconstruction, pancreaticoduodenectomy, and surgery for the gallbladder only) |
| CHOL | Cholecystectomy and gallbladder incision |
| COLO | Incision / excision or anastomosis of the large intestine. Including anastomosis of the large intestine and small intestine. Does not include rectal surgery. |
| ESOP | Surgery involving esophagectomy or reconstruction |
| GAST-D | Distal gastrectomy, Billroth-I or Billroth-II reconstruction |
| GAST-T | Total gastrectomy |
| GAST-O | Stomach incision or resection (excluding distal gastrectomy and total gastrectomy), not including vagal dissection and cardiaplasty |
| REC | Rectal surgery |
| SB | Incision or excision of the small intestine. Does not include small and large intestine anastomosis. |
| SPLE | Spleen resection or manipulation |
| XLAP | Abdominal surgery without operating the gastrointestinal tract or biliary system |
| We processed Japan nosocomial infections surveillance (JANIS) surgical classification criteria (available at: https://janis.mhlw.go.jp/english/index.asp. Accessed August 31, 2021.)  We excerpted surgical procedures related to gastrointestinal or hepatopancreatobiliary surgery. Translated in English by the author JO. | |
