# Supplementary material for: Severity of early diagnosed organ/space surgical site infection in elective gastrointestinal and hepatopancreatobiliary surgery
Source: Ann Gastroenterol Surg. 2021 Dec 21;6(3):445–53. doi: 10.1002/ags3.12539 (PMC9130879; doi:10.1002/ags3.12539)
Supplement: Supplementary file 5 — Table S2 [file AGS3-6-445-s001.docx]

**Supplemental Table 2. Organ/Space SSI criteria**

An organ/space SSI must meet the following criteria^1)^:

Infection occurs within 30 days after the operative procedure if no implant is left in place or within 1 year if implant is in place and the infection appears to be related to the operative procedure and infection involves any part of the body, excluding the skin incision, fascia, or muscle layers, that is opened or manipulated during the operative procedure

and

patient has at least one of the following:

a. Purulent drainage from a drain that is placed through a stab wound into the organ/space

b. Organisms isolated from an aseptically obtained culture of fluid or tissue in the organ/ space

c. An abscess or other evidence of infection involving the organ/space that is found on direct examination, during reoperation, or by histopathologic or radiologic examination

d. Diagnosis of an organ/space SSI by a surgeon or attending physician

1) HORAN, TC. Surveillance of nosocomial infections. Infect Control Hosp Epidemiol. 2004;1659–1702.
