# Supplementary material for: Severity of early diagnosed organ/space surgical site infection in elective gastrointestinal and hepatopancreatobiliary surgery
Source: Ann Gastroenterol Surg. 2021 Dec 21;6(3):445–53. doi: 10.1002/ags3.12539 (PMC9130879; doi:10.1002/ags3.12539)
Supplement: Supplementary file 6 — Table S3 [file AGS3-6-445-s008.docx]

| Supplemental Table 3. Types of organ/space SSI after elective surgery stratified by the surgical procedures | | | | | |
| --- | --- | --- | --- | --- | --- |
| Surgical Procedures | Overall Organ/space SSI cases | Anastomotic  leakage | Pancreatic fistula | Biliary fistula | Intra-abdominal abscess |
| ESOP | 19 | 17 | 0 | 0 | 2 |
| GAST-D | 10 | 1 | 5 | 0 | 4 |
| GAST-T | 1 | 0 | 1 | 0 | 0 |
| GAST-O | 2 | 2 | 0 | 0 | 0 |
| SB | 1 | 1 | 0 | 0 | 0 |
| COLO | 10 | 7 | 0 | 0 | 3 |
| APPY | 0 | 0 | 0 | 0 | 0 |
| REC | 11 | 7 | 0 | 0 | 4 |
| BILI-L | 14 | 1 | 1 | 10 | 2 |
| BILI-PD | 27 | 1 | 20 | 1 | 5 |
| BILI-O | 13 | 1 | 8 | 1 | 3 |
| CHOL | 2 | 0 | 0 | 1 | 1 |
| SPLE | 0 | 0 | 0 | 0 | 0 |
| Total | 110 | 38 | 35 | 13 | 24 |
| Data are reported as number of patients (% are calculated on the total patients in the line).  Surgical procedures are classified according to Japan nosocomial infections surveillance (JANIS) surgical classification criteria (See Supplemental Table 1).  SSI, surgical site infection. | | | | | |
