# Supplementary material for: Severity of early diagnosed organ/space surgical site infection in elective gastrointestinal and hepatopancreatobiliary surgery
Source: Ann Gastroenterol Surg. 2021 Dec 21;6(3):445–53. doi: 10.1002/ags3.12539 (PMC9130879; doi:10.1002/ags3.12539)
Supplement: Supplementary file 7 — Table S4 [file AGS3-6-445-s002.docx]

| Supplemental Table 4. Organ/space SSI after elective and emergency surgery stratified by the surgical procedures | | | | | | |
| --- | --- | --- | --- | --- | --- | --- |
|  | Keio University Hospital in 2016-2020  (Elective & emergency surgery) | | | JANIS in 2020  (Elective & emergency surgery) | | |
| Surgical Procedures | n | Organ/space SSI | Incidence | n | Organ/space SSI | Incidence |
| ESOP | 295 | 20 | 6.8% | 1,563 | 111 | 7.1% |
| GAST-D | 319 | 10 | 3.1% | 7,370 | 254 | 3.4% |
| GAST-T | 100 | 1 | 1.0% | 3,483 | 239 | 6.9% |
| GAST-O | 208 | 5 | 2.4% | 4,932 | 171 | 3.5% |
| SB | 325 | 5 | 1.5% | 7,171 | 220 | 3.1% |
| COLO | 697 | 12 | 1.7% | 46,193 | 1,150 | 2.5% |
| APPY | 97 | 2 | 2.1% | 13,411 | 186 | 1.4% |
| REC | 235 | 11 | 4.7% | 15,987 | 883 | 5.5% |
| BILI-L | 306 | 14 | 4.6% | 5,067 | 187 | 3.7% |
| BILI-PD | 190 | 27 | 14.2% | 3,167 | 562 | 17.7% |
| BILI-O | 317 | 13 | 4.1% | 3,419 | 376 | 11.0% |
| CHOL | 494 | 3 | 0.6% | 27,340 | 189 | 0.7% |
| SPLE | 19 | 0 | 0.0% | 301 | 9 | 3.0% |
| Total | 3602 | 123 | 3.4% | 68,692 | 2,392 | 3.5% |
| Data are reported as number of patients (% are calculated on the total patients in the line).  We processed Japan nosocomial infections surveillance (JANIS) data in 2020 (available at: https://janis.mhlw.go.jp/english/index.asp. Accessed August 31, 2021.)  Surgical procedures are classified according to JANIS surgical classification criteria (See Supplemental Table 1).  SSI, surgical site infection. | | | | | | |
