# Supplementary material for: Severity of early diagnosed organ/space surgical site infection in elective gastrointestinal and hepatopancreatobiliary surgery
Source: Ann Gastroenterol Surg. 2021 Dec 21;6(3):445–53. doi: 10.1002/ags3.12539 (PMC9130879; doi:10.1002/ags3.12539)
Supplement: Supplementary file 8 — Table S5 [file AGS3-6-445-s006.docx]

| Supplemental Table 5. Cause of death in organ/space SSI patients | | |
| --- | --- | --- |
|  | Organ/Space SSI Diagnosis date | |
|  | POD 1-4  (Early diagnosis group) | POD 5- (Normal-Late diagnosis group) |
| Number of total patients | 21 | 89 |
| Number of deaths 1 year after surgery | 5 (23.8%) | 9 (10.1%) |
| Cause of death |  |  |
| Death caused by organ/space SSI within  1 year after surgery | 3 | 3 |
| Cancer | 1 | 3 |
| Unexplained CPA | 0 | 2 |
| Pneumonia | 1 | 1 |
| Categorical values represent n (%).  CPA, Cardiopulmonary arrest; SSI, surgical site infection. | | |
